# Poor family functioning mediates the link between childhood adversity and adolescent nonsuicidal self‐injury

**DOI:** 10.1111/jcpp.12866

**Published:** 2018-01-24

**Authors:** Matthew Cassels, Anne‐Laura van Harmelen, Sharon Neufeld, Ian Goodyer, Peter B. Jones, Paul Wilkinson

**Affiliations:** ^1^ Developmental Psychiatry University of Cambridge Cambridge UK

**Keywords:** Family functioning, adversity, self‐harm, self‐injury, adolescence

## Abstract

**Background:**

Non‐suicidal self‐injury (NSSI) is a common harmful behavior during adolescence. Exposure to childhood family adversity (CFA) is associated with subsequent emergence of NSSI during adolescence. However, the pathways through which this early environmental risk may operate are not clear.

**Aims:**

We tested four alternative hypotheses to explain the association between CFA and adolescent‐onset NSSI.

**Methods:**

A community sample of *n* = 933 fourteen year olds with no history of NSSI were followed up for 3 years.

**Results:**

Poor family functioning at age 14 mediated the association between CFA before age 5 and subsequent onset of NSSI between 14 and 17 years.

**Conclusions:**

The findings support the cumulative suboptimal environmental hazards **(**proximal family relationships as a mediator) hypothesis. Improving the family environment at age 14 may mitigate the effects of CFA on adolescent onset of NSSI.

## Introduction

Around 17% of adolescents report non‐suicidal self‐injury (NSSI) (Swannell, Martin, Page, Hasking, & St John, 2014), any ‘deliberate and voluntary physical self‐injury that is not life‐threatening and is without any conscious suicidal intent’ (Laye‐Gindhu & Schonert‐Reichl, 2005). NSSI peaks in adolescence (Nock, 2010), and this is also the most common time of first incidence (Nixon, Cloutier, & Jansson, 2008). Adolescent NSSI is an important predictor of attempted/completed suicide (Wilkinson, Kelvin, Roberts, Dubicka, & Goodyer, 2011), and predicts the onset of later mental illness (Mars et al., 2014). It is therefore important to identify factors that can be the target of potential interventions for risk reduction in the incident rate of adolescent NSSI (Laye‐Gindhu & Schonert‐Reichl, 2005). Childhood family adversities (CFA) are particularly associated with the emergence of adolescent NSSI (Gratz, Conrad, & Roemer, 2002). The causal pathways and mechanisms for the well‐established association between CFA and subsequent NSSI up to two decades later are, however, unclear.

Two recent reviews concluded that although CFA was a robust predictor of NSSI, the roles of proximal adolescent factors, such as family functioning and mental illness, should be investigated as potential moderators or mediators of the association (Fliege, Lee, Grimm, & Klapp, 2009; Maniglio, 2011). Both reviews also emphasized the need for longitudinal analyses to clarify causal relationships between correlated risk factors for NSSI. We propose four possible models, supported by the literature.

### Mental illness model

Childhood family adversity is robustly associated with a range of deviant outcomes including subsequent conduct and emotional disorders, drug misuse, and mental illness (Dunn et al., 2011; Fergusson, Boden, & Horwood, 2008; van Harmelen et al., 2016). As higher prevalence of NSSI is associated with almost all mental illnesses (Dunn et al., 2011; Nock & Kessler, 2006), NSSI may not be a direct response or consequence of CFA itself but arise from subsequent mental illness.

### Suboptimal environmental hazards model

Childhood family adversity can negatively impact family functioning and child‐parent relationships (Hughes, 2004), and often occur within a context of more pervasive, chronic suboptimal family environments (Dunn et al., 2011). This continued and more proximal family dysfunction during adolescence may increase the risk for subsequent NSSI (Gratz et al., 2002) as opposed to the earlier experiences of CFA.

### Proximal environmental mitigation model

Adolescents’ primary sources of social support are parents and peers (Gottlieb, 1991). Proximal family relationships are more strongly associated with adolescent NSSI than are peer relationships (Hallab & Covic, 2010), despite the fact that adolescents who engage in NSSI are more likely to turn to their peers for support than to any other available source (Evans, Hawton, & Rodham, 2005). Moreover, having one strong source of social support may moderate the impact of dysfunctional relationships in another domain (Hazel, Oppenheimer, Technow, Young, & Hankin, 2014), and the combined effects of dysfunction in both family and peer relationships may be multiplicatively deleterious (Cyr, Clément, & Chamberland, 2014). Thus, positive family and/or peer environment in adolescence would reduce the effect of CFA (Aspelmeier, Elliott, & Smith, 2007; Collishaw et al., 2007), either because the adolescent would have found social supports from their positive family and/or peer interactions, or because the family members who previously contributed to adversity are now more positive supports.

### Attachment model

Conversely, attachment theory would suggest that CFA is a necessary and sufficient cause for later psychopathology including NSSI as early experiences inform an immutable internal working model of the world (Bowlby, 1988). This hypothesis predicts that schemas formed in the context of CFA would lead to appraising the world as hostile in adolescence and later life. As there would be no updating of the internal model, these schemas would persist regardless of more proximal experiences such as improved family functioning in adolescence. Thus, there would be a main effect between CFA and the emergence of NSSI in adolescence, and no interaction with/mediation by more proximal factors.

We tested these four hypothesized models of the intermediate pathway between CFA (i.e. pre‐age five) and adolescent (between ages 14 and 17) onset of NSSI:

1. Mental illness model: mental illness before the age of 14 as a mediator
2. Suboptimal environmental hazards model: proximal family relationships as a mediator
3. Proximal environmental mitigation model: proximal family and/or peer relationships as a moderator
4. Attachment model: a direct relationship that is neither mediated nor moderated by proximal family relationships.

## Methods

### Participants and procedures

Data for this study were collected as part of Roots, a larger longitudinal study of risk factors for the development of psychopathology. Roots is a community sample of adolescents recruited from a wide geographical area extending 30 miles north, 20 miles south and 20 miles west of Cambridge United Kingdom (Goodyer, Croudace, Dunn, Herbert, & Jones, 2010; Lewis, Jones, & Goodyer, 2015). In total, 27 secondary schools (25 state and two private schools) were approached for participation, of which 18 agreed. Through these schools, 3,762 students were invited to participate. Overall, consent forms were received from 1,238 (33%) students; 675 girls (54.5%), and 563 (45.5%) boys. Although the sample covers a wide socioeconomic range as measured by ACORN (see below), the sample is disproportionately affluent, comprising roughly twice as many ‘wealthy achievers’ and only half as many participants of ‘moderate means’ or ‘hard‐pressed’ families compared to UK figures. The study was approved by Cambridgeshire two research ethics committee, reference number 03/302. Data were collected when the adolescents were 14‐, 15.5‐, and 17‐year old.

Since our primary outcome variable is new onset of NSSI by age 17, all analyses were performed on subsample of 933 participants who reported no lifetime NSSI by age 17 and provided follow up data on NSSI at age 17. Thus, all instances of NSSI reported at age 17 happened between ages 14 and 17 and all observed associations between other variables and NSSI are prospective.

### Measures

#### Drug, alcohol and self‐injury questionnaire (DASI)

The DASI was developed as a self‐report measure of cigarette, alcohol and drug use and NSSI. Our primary outcome variable was a binary question: ‘Have you ever tried to hurt yourself on purpose without trying to kill yourself?’ This question was applied at the 14 and 17 year assessments. We have demonstrated some reliability and validity of this question through finding similar population prevalence of NSSI in two separate community studies, and moderate convergent validity (*r* = .66) with the total score of another well‐validated multi‐item measure of self‐harm behavior, the Self‐Harm Inventory (Sansone, Wiederman, & Sansone, 1998) in a third community sample of 700 adolescents (ages 16–18) from the Cambridgeshire area (further details available from 1st author). Using a single‐item measure of NSSI is common in NSSI research and has previously been shown to render consistent estimates of prevalence (Muehlenkamp, Claes, Havertape, & Plener, 2012).

#### Cambridge early experience interview (CAMEEI)

The CAMEEI (Dunn et al., 2011; St Clair et al., 2015) was used to measure childhood family adversity (CFA). The CAMEEI is a semi‐structured interview conducted with the adolescent's primary caregiver. The interview comprises questions about several components of CFA, including abuse, family discord, family loss, parental mental illness and parenting style over three distinct age epochs (pre‐primary school; primary school; secondary school).

In a prior analysis, factor analysis did not suggest a unidimensional structure of exposure to these adversities, and model fit was poor for the two factor model (Dunn et al., 2011). We therefore used a mixture model perspective, which grouped individuals by their experience of multiple adversities using latent class analysis (LCA). LCA identified four patterns of early family environment in our participants before the age of 5: Optimal class, with low levels of adversity (69%); Discordant class, with moderate levels of adversity (19%); Hazardous class, with high levels of adversity (6%); Atypical parenting class, with low levels of adversity but high levels of suboptimal parenting and moderate levels of low maternal warmth (7%). These latent classes are orthogonal rather than ordinal. To enable the proposed analysis to have adequate power, and to be consistent with other analyses from Roots, participants were dichotomized. As the latter three classes, all reflect suboptimal early family environment, participants from these three classes were combined into a ‘childhood family adversity’ (CFA) present group, while those from the optimal class were classified as no CFA.

#### The Kiddie‐SADS‐Present and Lifetime Version (K‐SADS‐PL)

The K‐SADS‐PL (Kaufman et al., 1997) was used to assess whether participants met DSM‐IV criteria for the diagnosis of a mental disorder. The K‐SADS‐PL is a semistructured interview about participants’ current and previous experiences of psychopathology. The participant and a caregiver were interviewed separately when the participant was age 14. A clinical diagnosis was then assigned by a consultant psychiatrist. In two longitudinal studies, adolescents with ‘High Clinical Index (HCI)’ case status (one symptom less than threshold, in conjunction with significant impairment) showed similar psychopathology trajectories to those meeting full diagnosis of major depression (Fergusson, Horwood, Ridder, & Beautrais, 2005; Johnson, Cohen, & Kasen, 2009). Therefore, consistent with other analyses from Roots, participants were dichotomized based on presence or absence of a diagnosis/HCI of any mental disorder (depression, anxiety disorders, eating disorders, substance use disorders, and disruptive behavior disorders) at or before age 14.

#### The McMaster Family Assessment Device General Functioning Subscale (FAD‐GF)

The FAD‐GF (Epstein, Baldwin, & Bishop, 1983) was used to evaluate current general family functioning at the age of 14. The FAD‐GF consists of 12 self‐report questions about the overall current quality of family relationships. Higher FAD‐GF scores are associated with better family functioning. The FAD‐GF is widely used and its psychometric reliability and validity have been demonstrated in a number of samples (e.g. (Kabacoff, Miller, Bishop, Epstein, & Keitner, 1990).

#### A classification of residential neighbourhood

A Classification of Residential Neighbourhood (ACORN) (CACI Information Services, 1997) was used as a measure of socioeconomic status (SES). Five levels of SES (Wealthy Achiever, Urban Prosperity, Comfortably off, Moderate means, Hard‐pressed) were derived from postcodes (http://www.caci.co.uk). In this study the sample was dichotomized as belonging to moderate means/hard pressed (low SES) versus any of the more affluent categories.

#### The Cambridge Friendship Questionnaire (CFQ)

The CFQ (Goodyer, Wright, & Altham, 1989) was used to measure the quality of children's relationships with their peers. The CFQ is an eight item self‐report instrument that assesses the number, availability, and quality of friendships. The CFQ was developed from a semi‐structured interview based on ethological principles of social relationships and the developmental significance of friendships (Pellegrini & Bartini, 2000). The CFQ has demonstrated good reliability and validity (α = .70, further details available from first author), and has demonstrated ecological validity across two samples (van Harmelen et al., 2016, 2017). The CFA yields a single total score with higher scores indicating more positive perceptions of peer relationships (i.e. ‘Friendships’).

### Statistical analysis

**Figure 1:**
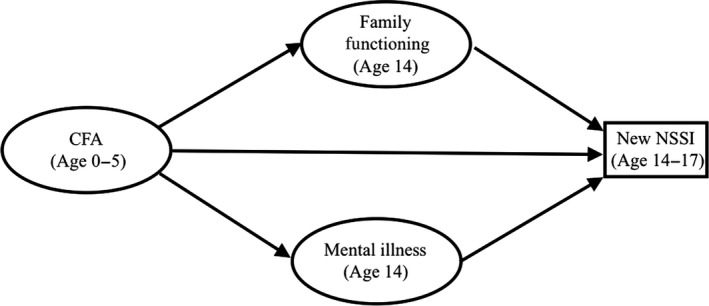
Path diagram of the proposed model of potential pathways from childhood family adversity (CFA) to new NSSI by age 17, both directly and through family dysfunction at age 14 and diagnosis of mental illness before age 14

We set out to investigate the relationship between CFA (<5 years) and adolescent onset of NSSI (between age 14 and 17) through two separate mediator pathways (i.e. family relationships at age 14 and mental illness up to age 14) shown in Figure 1. We also tested whether family and peer relationships moderated the effects of CFA, using CFA x family/peer relationships interaction terms. We used the user‐written binary logistic mediation package (Ender, 2011) for STATA. Robust confidence intervals for direct and indirect effects were estimated using 5,000 bootstrap repetitions. The binary mediation package does not provide *p* values.

Prior to running a multiple mediation model, we examined the correlations between NSSI by age 17 and potential predictor variables, including family and peer relationships at age 14 and mental illness pre‐14. To aid interpretation and comparisons, *r* statistics were calculated: point‐biserial correlations for dichotomous‐continuous associations, and tetrachoric correlations for dichotomous‐dichotomous associations.

As there were significant predictors of missingness in our baseline data, data cannot be presumed to be missing at random, potentially biasing estimates (Sterne et al., 2009). In the subsample of participants without lifetime NSSI at age 14 (our inclusion criteria), there was minimal missing data on NSSI at age 17 (12%, see results for more details). However, with the inclusion of baseline model variables, missingness in follow up data increased to 26% (*n* = 781), reducing the number of new NSSI cases from 59 to 47. Therefore, we performed multiple imputation of baseline model variables using chained equations, producing 17 imputations (our variable with greatest missingness, family functioning, was missing for 17% of our sample). Sixteen significant predictors of our primary variables (pre age 14 mental illness, age 14 family functioning, pre age 5 CFA, and new onset NSSI by age 17), and missingness on these variables were included in our imputation model. Analyses were performed on imputed data based on complete cases of NSSI at age 17, and no reported lifetime engagement in NSSI by age 14.

Analyses were conducted using STATA, version 14 (StataCorp, 2015). A threshold of 5% was used for statistical significance, as predictor variables were correlated and only one primary outcome variable was used.

## Results

### Univariate associations with NSSI and CFA

At age 14, 1,202 participants reported on NSSI, of which 1059 (88%) reported no history of NSSI. Of this latter group, 933 (88%) participants reported on NSSI up to age 17. A total of 59/933 (6%) participants reported new onset of NSSI by age 17. Table 1 demonstrates univariate associations between our predictor variables and new onset of NSSI between the ages of 14 and 17. Poorer family functioning at age 14, and mental illness by 14, and CFA were positively associated with new onset of NSSI between ages 14 and 17. Friendships and SES were not associated with NSSI. Gender was also not associated with new onset of NSSI from ages 14–17 (male new incidence = 0.05%, female new incidence = 0.07%, χ^2^ = 1.06, *p* = .35).

**Table 1:** Correlations between new NSSI from ages 14 to 17, CFA, and potential mediator and explanatory variables measured at the age of 14

	New Onset of NSSI		CFA	
	*r*	*p*	*r*	*p*
CFA	.08	.020		
Gender	.03	.303	−.05	.100
Diagnosis	.07	.037	.18	<.001
SES	−.01	.729	.17	<.001
Family functioning	−.09	.007	−.10	.001
Friendships	−.05	.173	−.06	.071

CFA, childhood family adversity; NSSI, Non‐suicidal self‐injury; SES, socioeconomic status.

*r* statistics represent tetrachoric correlations for CFA, gender, DSM diagnosis, and SES, and point‐biserial correlations for family functioning and friendships.

Table 1 also shows correlations between our predictor variables and CFA. Poorer family functioning at age 14 and diagnosis of a mental illness before age 14 were positively correlated with CFA and new NSSI. Therefore, family functioning and mental illness are potential mediators for the CFA‐NSSI association. Family functioning and mental illness were reasonably uncorrelated with each other (*r* = −.12) and had low variance inflation factors (mean variance inflation factor = 1.01), suggesting that multicollinearity was not an issue in our model.

### Revealing a psychosocial model for 1st episode NSSI

**Figure 2:**
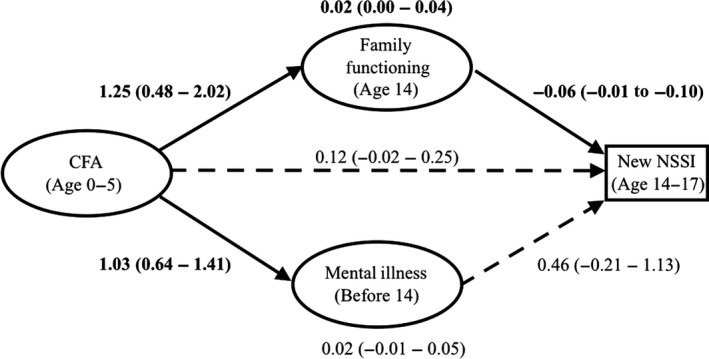
Path diagram of the multiple mediation model of the effect of CFA on new onset of NSSI through mental diagnosis and family functioning. The model displays standardized coefficients (95% confidence intervals) of the direct effects of CFA and mediators on NSSI; and the indirect effects of CFA on NSSI through each of the mediators (at the top and bottom of the figure). Significant effects (*p* < .05) are shown in bold with solid lines

Results of the binary logistic multiple mediation analysis are shown in Figure 2. Family functioning significantly mediated the association between CFA and NSSI. The direct pathway between CFA and NSSI was nonsignificant as was the indirect pathway through mental illness before age 14. This model accounted for 16% of the variance in new onset of NSSI between aged 14 and 17. Friendships and family functioning did not significantly moderate the effects of CFA or mental illness on NSSI, nor did friendships moderate the effects of family functioning on NSSI (all *p* > .08).

Findings with complete case analyses resembled those with imputed data, however in this smaller sample of 783 participants with complete data, the indirect pathway from CFA to NSSI through mental illness was also significant (estimate = −0.02, CI = −0.05–0). As with the imputed data, in full case analyses the indirect path from CFA to NSSI path through adolescent family functioning was also significant (estimate = −0.04, CI = −0.01 to 0.09).

## Discussion

We tested four hypothesized models of the association between early childhood family adversity (CFA) and the onset of non‐suicidal self‐injury (NSSI) between the ages of 14 and 17. We found evidence to support the chronic suboptimal environmental hazards hypothesis: family functioning at age 14 mediates the association between CFA before the age of 5 and onset of NSSI between ages 14 and 17. When traumatic experiences happen in the context of continuing family dysfunction, are perpetrated by a parent figure, or are not responded to adequately by a parent, family functioning is likely to be impaired (Hughes, 2004). Impaired family functioning later in adolescence is in turn robustly associated with NSSI (Gratz et al., 2002). Family functioning may influence adolescent NSSI through several risk factors for NSSI such as impulsivity, emotion regulation (Scott, Levy, & Pincus, 2009), self‐esteem (Collins & Read, 1990), interpersonal skills (Hazel et al., 2014), coping skills, and mental illness (Moretti & Peled, 2004). These pathways warrant further investigation. The present findings, however, suggest that improving family relationships may reduce the later onset of NSSI in children who have been exposed to CFA.

The indirect pathway from CFA to NSSI through mental illness was not significant. It is worth noting that in our complete case analyses this pathway was significant along with the pathway through family functioning, supporting the mental illness model. Therefore, it is possible that treating mental illness may mitigate some of the effects of CFA on adolescent NSSI. The nonsignificant finding in the imputed cases analysis may be a type 2 error; alternatively the significant finding in the completed cases analysis may be due to attrition bias, corrected by the imputation. Further analysis in larger datasets is warranted to answer this important question.

We found no support for our proximal environmental mitigation model: positive proximal peer and family relationships do not reduce the effects of CFA, mental illness, or poor peer/family relationships on the incident risk rate of NSSI between 14 and 17 years. Indeed, peer relationships did not affect risk of NSSI, in keeping with the literature (Hallab & Covic, 2010). With regard to family relationships, whether or not family adversity continues seems to be the primary factor that influences risk of NSSI, rather than support from positive family members reducing harm from earlier adversity. Furthermore, we found no support for our attachment model: there was no significant direct association between CFA and adolescent‐onset NSSI, when mediating effects of proximal family adversity and mental illness were controlled for. This suggests that the effects of CFA on NSSI are modifiable and perhaps that internal working models of threat can be updated by subsequent experience.

### Clinical implications

Findings from this study are consistent with a large amount of pre‐existing literature demonstrating that CFA has long‐term psychopathological consequences (Perry, Pollard, Blaicley, Baker, & Vigilante, 1995) including being a risk factor for adolescent NSSI (Maniglio, 2011). Reducing CFA, therefore, is likely to reduce NSSI. However, the present findings also suggest improving family function after CFA may reduce later NSSI. It is important, therefore, that services that help families in trouble, such as social care, try to address family relationships directly. Further research is needed to investigate potential methods of improving family relationships after CFA. Future studies should then examine whether these improvements reduce later NSSI. However, the present findings do support a family‐focused approach to preventing adolescent NSSI. One such approach, Attachment Based Family Therapy (ABFT) (Diamond, Reis, Diamond, Siqueland, & Isaacs, 2002), which focuses on improving communication and support in child‐parent relationships, has been shown to be effective among adolescents at reducing both depression and suicidality (Diamond et al., 2010). As depression and suicidality are both closely related to NSSI (Wilkinson et al., 2011), the efficacy of ABFT on reducing the risk of NSSI seems theoretically promising and should be investigated with larger randomized control trials.

### Limitations

One weakness of this study is that the single‐item measure of NSSI, ‘Have you ever tried to hurt yourself on purpose without trying to kill yourself?’ may not have been sufficient to capture all NSSI acts and did not distinguish between different methods, motivations or frequencies of NSSI. This is potentially problematic as different methods and frequencies of NSSI have been related to different psychological and environmental factors (Rodham, Hawton, & Evans, 2004). However, with a sample size of less than 1000, we would not have had sufficient power for mediation/moderation analyses if we had split our primary outcome variable. Moreover, ‘trying’ to hurt oneself is not precisely the same as definitively ‘hurting’ oneself, and therefore the item may more accurately measure NSSI intent as opposed to behavior. Nevertheless, the NSSI item used in this study showed adequate agreement with a well‐validated measure of NSSI in a similar population.

Another limitation of this study is that the sampling age range may have been too late to capture many first incidents of NSSI. The natural course of NSSI is curvilinear, with a sharp increase around age 12 and a decrease in later adolescence (Plener, Schumacher, Munz, & Groschwitz, 2015). Longitudinal studies beginning at a younger age (before 12) would be greatly beneficial as they would capture more first incidents of NSSI and therefore have greater statistical power for detecting prospective risk factors.

A final weakness of this study was that CFA was assessed retrospectively at the age of 14, which may have reduced accuracy (Ebner‐Priemer et al., 2006).

**Key points**

- Childhood family adversity (CFA) is robustly associated with adolescent non‐suicidal self‐injury (NSSI).
- Pathways between CFA and subsequent onset of NSSI are unclear.
- We demonstrate that the association between CFA before age 5 and NSSI between ages 14–17 is mediated by poor family functioning at age 14.
- These findings suggest that improving family function after CFA may prevent later NSSI.
- These findings support a family‐focused approach to preventing adolescent NSSI.
